# The care capacity goals of family carers and the role of technology in achieving them

**DOI:** 10.1186/s12877-020-1455-x

**Published:** 2020-02-27

**Authors:** Myles Leslie, Robin Patricia Gray, Jacquie Eales, Janet Fast, Andrew Magnaye, Akram Khayatzadeh-Mahani

**Affiliations:** 1 Department of Community Health Sciences, Cumming School of Medicine, University of Calgary, Calgary, Alberta Canada; 2 School of Public Policy, University of Calgary, Calgary, Alberta Canada; 3 Department of Human Ecology, University of Alberta, Edmonton, Alberta Canada; 4 Health Services Management Research Center, Institute for Futures Studies in Health, Kerman University of Medical Sciences, Kerman, Iran

**Keywords:** Family caregivers (FCs), Elderly, Aging population, Sustainability, Resilience, Quality of care, Technology

## Abstract

**Background:**

As global populations age, governments have come to rely heavily on family carers (FCs) to care for older adults and reduce the demands made of formal health and social care systems. Under increasing pressure, sustainability of FC’s unpaid care work has become a pressing issue. Using qualitative data, this paper explores FCs’ care-related work goals, and describes how those goals do, or do not, link to technology.

**Methods:**

We employed a sequential mixed-method approach using focus groups followed by an online survey about FCs’ goals. We held 10 focus groups and recruited 25 FCs through a mix of convenience and snowball sampling strategies. Carer organizations helped us recruit 599 FCs from across Canada to complete an online survey. Participants’ responses to an open-ended question in the survey were included in our qualitative analysis. An inductive approach was employed using qualitative thematic content analysis methods to examine and interpret the resulting data. We used NVIVO 12 software for data analysis.

**Results:**

We identified two care quality improvement goals of FCs providing care to older adults: enhancing and safeguarding their caregiving capacity. To enhance their capacity to care, FCs sought: 1) foreknowledge about their care recipients’ changing condition, and 2) improved navigation of existing support systems. To safeguard their own wellbeing, and so to preserve their capacity to care, FCs sought to develop coping strategies as well as opportunities for mentorship and socialization.

**Conclusions:**

We conclude that a paradigm shift is needed to reframe caregiving from a current deficit frame focused on failures and limitations (burden of care) towards a more empowering frame (sustainability and resiliency). The fact that FCs are seeking strategies to enhance and safeguard their capacities to provide care means they are approaching their unpaid care work from the perspective of resilience. Their goals and technology suggestions imply a shift from understanding care as a source of ‘burden’ towards a more ‘resilient’ and ‘sustainable’ model of caregiving. Our case study findings show that technology can assist in fostering this resiliency but that it may well be limited to the role of an intermediary that connects FCs to information, supports and peers.

## Background

Canada’s population is aging, with a recent estimate predicting that 21% of residents will be over the age of 65 by 2040 [1]. As elsewhere, this demographic trend has brought with it both an increase in the prevalence of chronic diseases and disabilities, and a shift on the part of health care systems from acute to community-based care delivery. In the context of this shift, Family Carers (FCs) have become central to governmental efforts to reduce demands on formal health and social care systems [2–6]. The Family Caregiver Alliance defines a FC as “any relative, partner, friend or neighbor who has a significant personal relationship with, and provides a broad range of assistance to an older person or an adult with a chronic or disabling condition.” This expansion of the concept of ‘family’ reflects the demographic reality of Canadian families which have steadily decreased in size over the past three decades [7], and become more geographically dispersed, leaving the care of elders to fewer children and proximate family members and friends. In Canada, in 2012, two-thirds of 8.1 million FCs provided care to an older adult above 65. These 5.4 million FCs included not only adult children caring for parents, but also people caring for grandparents, spouses, extended kin, friends and neighbours [8]. In fact, 28% of Canadians over the age of 15 are FCs [9] and nearly half (46%) have been a caregiver at some point in their life already. The gender gap in caregiving is closing, with women comprising 54% of all caregivers to older adults in Canada and men comprising 46%. Yet, most of the demanding and intense care work continues to fall predominantly to women, as is the case elsewhere in the world [10–12]. The monetary value of FC’s unremunerated work is estimated at $66.5 billion annually [13].

The demands associated with this unpaid work come with both rewards [14–18], and heavy consequences [9, 19–23]. While FCs derive satisfaction from their work and strengthened bonds with care receivers, the negative consequences of caregiving are well-documented [24, 25] and include truncated social networks [23], reduced or limited labour force participation [26], and poorer physical [27] and mental health [21, 28, 29]. With one-third of FCs in a recent study spending an average of 20 h a week caregiving [30], it is perhaps unsurprising that social engagement and paid labour force participation suffer as the risk of physical and mental health problems increases [30, 31]. Evidence suggests that the majority of employed FCs experience difficulty juggling their paid work and caregiving responsibilities [12, 32] with negative impacts on their ability to sleep, their productivity at work and their peace of mind concerning care receivers [33–35]. Many FCs miss days of work because of their caregiving, and some reduce their paid work hours or exit the labour force as they struggle to balance competing responsibilities [13, 36].

In this context, improving the sustainability of unpaid care work [37] has become an area of intense interest. FCs view caregiving as sustainable when they are able to strike a favourable balance between the rewards and demands of caregiving [37] amidst their other competing responsibilities. Technology-based interventions are showing the potential to support FCs caring for older adults – especially those caring for persons with dementia [38–40]. For instance, internet-based monitoring systems -- consisting of cameras and sensors and aimed at improving the safety of older adults with dementia as they continue to live at home – have improved outcomes for FCs. Specifically, researchers found that the extended free time and peace of mind experienced by these FCs helped improve their quality of life [41]. In another study, GPS tracking technology was shown to help people in the early stages of dementia and their FCs feel less stressed and worried [42]. As such, many policy makers and commercial developers have come to see technology as a panacea that will enhance the sustainability of FCs’ caregiving [43–45].

This paper focuses on understanding the holistic goals of FCs who provide care to older adults, and how these goals do, or do not, link to FCs’ views on technology as a potential support mechanism. The principal questions underlying our research were: what are the key goals of FCs providing care to older adults and how might technology assist in achieving those goals? While there is a growing body of research addressing the effectiveness and feasibility of technology-based interventions to support FCs caring for older adults [38–40, 46, 47], little is known about FCs’ perspectives about how technology can assist in making their care work more sustainable. Our research aimed to address this knowledge gap.

We present the results of a thematic content analysis of qualitative data from a mixed-methods study. The first data are from focus group conversations, and the second are from open-ended responses to an online survey. We present an account of the personal goals that FCs espouse when they imagine improving the care they themselves provide. Combining our focus group and survey data, we show how technology is, or is not, implicated in carers’ aspirations to enhance their capacity to provide quality care. As we present our data, we show that, in seeking to achieve their personal care quality goals, FCs are simultaneously seeking to improve their resilience and so render their care work sustainable.

## Methods

### Study design

To better understand the FCs’ (caring for older adults) goals of personally providing the best care possible and how technology might support them in achieving those goals, we employed a sequential mixed-method approach using qualitative focus groups and an online survey. The focus groups were conducted first. Findings from the focus groups were then used to inform the design of the online survey. As both the focus groups and the survey aimed explicitly at tapping into FC’s goals, we merged the data for analysis. Our rationale for adopting a goals-based approach was that it avoided many of the pitfalls of focusing on needs by encouraging participants to think into the future and about how technology might facilitate not just task-related efficiencies of ‘doing’ care, but also improvements in navigating relationships in the context of care [25]. This approach encouraged participants to focus on the role technology might play in supporting specific tasks *and* relationships. A key objective in merging the data sources was to identify points of concordance and divergence across the two different data collection modalities. In this way, we used the two sources to cross validate one another and arrive at a more robust saturation within our analytic categories. We describe the two data collection modalities in detail below.

### Focus groups

We conducted 10 face-to-face focus groups in Alberta, Canada from May 2017 to August 2018. Focus groups are exploratory moderated group discussions with the aim of drawing out the perceptions, attitudes, and experiences of participants with common interests in regards to a specific phenomenon [48] – in our case, family care to older adults.

The eligibility criteria for FCs to participate in our focus groups were: 1) self-identify as a caregiver to an adult over the age of 65; 2) be less than 65 years old (our assumption was that this age group would be more likely to adopt new technology); 3) speak English; 4) reside in the Calgary or Edmonton areas (driven by the costs and time associated with travel); 5) preferably be working in the paid labour market, either part-time or full-time (again, our assumption was that this demographic would be more likely to adopt new technology) [49].

To recruit FCs into our focus groups, we employed a mix of convenience/opportunistic and snowball sampling strategies. As FCs constitute a heterogeneous population [50], their identification and recruitment in research is difficult [51–56]. Drawing on the literature [49] we employed multiple strategies to recruit FCs including: 1) collaborating with community-based carer organizations such as Alzheimer Society, Caregivers Alberta, yielding 13 FCs, 2) distributing study flyers in two geriatric clinics affiliated with the University of Calgary yielding only 2 FCs, 3) advertising through social media (e.g. Facebook and Twitter) as well as print media (e.g. Dementia Connections magazine) yielding 9 FCs, and 4) traditional snowball sampling by asking recruited FCs to encourage those who might be interested to participate in our research, which yielded only one FC [49].

Three to five FCs participated in each two-hour focus group. In total we recruited 25 FCs of whom 21 were female and 4 were male. All were under 65 caring for an older adult above the age of 65; two FCs (both females) were less than 30 years of age and the rest were between 30 and 65 year old. Of 25 FCs, 10% were working full time, 50% working part-time, and the rest were not working. Three FCs were from Cochrane (a town located 18 km west of the Calgary city), five were from Edmonton and the rest were from Calgary – the latter two being large census metropolitan areas.

Each FC participated in two focus groups held at least 1 week apart. In the first session they discussed their goals and then imagined potential technology solutions in the second. All focus groups were digitally recorded and transcribed verbatim. This phase of research was approved by the University of Calgary and University of Alberta Research Ethics Boards. Written informed consent was obtained from all participants at the beginning of each focus group.

### Survey

Based on the findings from the focus groups and building on the research literature about the impact of caregiving on FCs’ lives, we developed an online survey aimed at enhancing FCs’ well-being. The main objectives of the survey were to: 1) investigate FCs’ goals in seven domains (physical health; mental health and well-being; social connections; education; employment; financial well-being; and care work); and 2) examine FCs’ beliefs about the potential role of technology in helping them achieve their goals.

The survey included three blocks of questions. In the first block, respondents were asked to state how important a short list of goals for each of the seven key domains were to them in their care journeys. The second block of survey questions centered around the current role that digital technology played in their everyday lives, their beliefs about these particular tools and their ideas for innovative products and services that might help them better meet their goals to enhance their well-being. The final block of survey questions collected socio-demographic and care history information from respondents. At the end of block 2, we asked an open-ended question: “As a caregiver, I wish I had …” . This question was intended to afford FCs the opportunity to make any additional comments beyond what was captured in the survey questions. Only responses to this open-ended question were merged with focus group data and included in our qualitative data analysis.

To recruit FCs to the online survey we worked through partner organizations such as the Canadian Caregiver Network, CARP (the Canadian Association of Retired Persons), Carers Canada, Caregivers Alberta, Early Onset Dementia Alberta, the Canadian Home Care Association, Young Carers Initiative, CapitalCare Edmonton, Vanier Institute of the Family, and the University of Alberta Department of Human Ecology. The Human Research Ethics Board 2 at the University of Alberta approved this phase of the project.

The survey was completed by 599 FCs from across Canada, with the largest group residing in Ontario (38.1%), followed by British Columbia (19.5%), Alberta (18.6%) and Quebec (13.7%). The remaining provinces and territories accounted for 7.7% of the sample. Of survey participants, 77% were women and 21% were men. Five percent (5.4%) were under the age of 35 years old, 23.2% were between the ages of 36 and 55 years old, 26.2% were between the ages of 56 and 64 years old, 30.7% were between the ages of 65 and 74 years old and 12.8% were over the age of 75 years old.

The majority of our FCs were retired (45.1%) or employed (32.5%); 6.8% were self-employed, 2.5% were looking for work and 2.5% were currently students. Less than 9 % (8.9%) of survey participants reported other forms of productive work including semi-retired, providing care full-time, and on work-related leave or absences.

### Data analysis

An inductive approach [57] with thematic content analysis methods were employed to examine and interpret the qualitative focus group and survey data. Thematic content analysis is defined as “a method for identifying, analysing and reporting patterns (themes) within data” ([58]: p79). There are different approaches to thematic content analysis and the one we adopted has been described as conventional qualitative content analysis (as opposed to ‘directed’ or ‘summative’ approaches). Our inductive data analysis was completely data-driven using a grounded theory approach with neither a priori codes derived from theory (as in the directed approach) nor counting words/terms (as in the summative approach) used [59]. We took the following steps in our analysis: 1) familiarizing ourselves with the data; 2) generating the initial codes; 3) revisiting the initial coding; 4) searching for categories and patterns; 5) developing an initial list of categories; 5) reviewing and modifying the categories; and 6) revising the categories and sub-categories [58, 60]. The data analysis involved one author reviewing the verbatim transcripts and free form survey responses and assigning initial codes and sub codes inductively to form emergent concepts. These codes were reviewed and refined through constant comparison and discussion with three other co-authors until consensus was reached. Raw data were read, sorted, and re-read repeatedly searching for categories and patterns. Through an iterative process of discussions, the distinctions between the categories and patterns became clearer [61]. The coding framework was finalized once no new concepts emerged. NVIVO 12 software was used to support coding data and developing categories.

## Results

FCs caring for older adults expressed, in our research, two principal goals, specific to their care-related work. These focused on: 1) enhancing and 2) safeguarding their capacity to care. To enhance their capacity to provide quality care, FCs sought: i) better foreknowledge of their care recipient’s condition; and ii) an improved ability to know about and navigate existing support systems. There appeared to be only limited space for technology to assist in enhancing the capacity to know and be prepared for future changes in their care recipient’s condition. The desire for a more navigable system was seen as more amenable to technological intervention, with FCs envisioning applications that could help them find and maneuver through an otherwise unconnected and incoherent ‘system’ of supports, programs, insurers, and providers. To safeguard their own wellbeing, and so to improve resilience and make their care work sustainable, FCs sought improved self-care skills that included better coping strategies and opportunities for mentorship, and socialization. Technology was seen as well positioned to help achieve better self-care and to facilitate the connections required for mentorship and a richer social life.

### Enhancing care capacity: seeking a crystal ball

Participants sought foreknowledge of what would happen next in their loved one’s disease trajectory. FCs believed that this insight had the potential to improve both the care interventions they undertook and their subjective well-being over the long term. As one survey respondent described it, he was looking for:

Better resources to describe “what happens next” even “might happen” as the disease (Alzheimer’s dementia) eats away at my wife’s brain. **SR**^1^ **16**

Another FC described wanting “An idea of what my mom will need in the future so I can better plan now,” **SR228**. A focus group participant gave a more detailed account of what she was looking for to enhance her capacity to provide care into the future:

I want to know if the [medically identified phases of dementia] are the same for a 60-year-old with early onset as [they are for] an 80- or a 90-year-old. [Is my care receiver] going to go from this phase to that [phase] much faster? [I’m looking for] a crystal ball. **FG1R2**

Others used the same formulation, expressing a desire for:

...a crystal ball so I know what the future might look like, so I can prepare more for what might be required as we go forward. **SR411**

For some, the metaphor of the crystal ball was used exclusively to enhance their capacity to provide care. One FC described this desire to know the future:

I kind of have an idea what’s coming. I just don’t know when it’s going to happen. I’m constantly looking up to see: “Is this normal? Can I expect this? Do I have to call the doctor?” **FG6R1**

Other FCs, however, saw prescience as a tool for managing their own emotions and well-being rather than the needs of their loved one alone. A survey respondent described her search for:

A crystal ball to see [into] our time left together [and so] better plan for the future and manage my expectations. I believe the definition of “happiness” is the successful management of one's expectations. **SR324**

Rather than seeing foreknowledge as a route to more appropriate and better-timed interventions for her loved one, this FC, and others, saw prescience as an aid in dealing with the emotions and burden of caregiving. It would, they hoped, provide them with an improved capacity to manage not just their expectations, but also their own well-being, helping to avoid anger at having made what they saw, in retrospect, as the wrong choices. As one FC noted:

As a caregiver I am angry that I [chose] independence when it turned out I had such a short time frame with the one I loved. **SR358**

FCs, who identified foreknowledge as a goal, believed that it would enhance both the quality of care they provided and the timeliness with which they accessed outside services. It would also improve their own resilience by allowing them to manage their expectations, anger, and the other burdens of caregiving.

Although knowing “what’s next” was a central aspiration for the vast majority of those participating in our survey and focus groups, most were unable to put forward ideas as to how technology might solve this quintessentially human desire to see into the future. Those who did have suggestions imagined internet-based chat rooms for the exchange of information. One FC wished for an app that would:

connect to specific health issues and the hospital care team. With those health issues, maybe there’s a chat room, and [in that chat room you can learn] “these are the things you could expect.” **FG2R1**

In this sense, the FC is imagining the internet as a community-building tool in which health professionals, and potentially other lay people, can get a better idea of what the future holds in the context of a specific disease.

### Enhancing care capacity: navigating the ‘system’

A second goal FCs had to enhance their capacity to care saw FCs seeking more integrated and comprehensive knowledge, not of the future, but of the present state of supports and programs that were available to them. One FC summed up the experience of many when she described feeling lost in the broad range of available supports and their eligibility requirements. She noted, “There are so many different things, and you don’t know where to go,” (**FG4R1**). Another hoped for “a better roadmap of all the supports available to me” (**SR41)**. Such a roadmap would help FCs “navigate the system and not feel in the dark,” guiding them on their “path as a caregiver within the context of [their] personal circumstances” (**SR135**). It would also help eliminate the sense that random chance and luck had led them either to, or away from, a particular constellation of supports and programs.

My goal would be to be more connected to all the supports and agencies available … I always feel like I’m bumping around, and I luck out and I find this, and I luck out and I find that resource. (**FG7R3)**

In this way, access to a comprehensive repository of supports, tailored to personal circumstances that would allow for individual ‘system’ navigation, was a central goal for FCs. Achieving this goal would bring them out of the dark, at once enhancing the care they could provide to their care receiver, and improving their own mental outlook and resilience in the process.

FCs’ proposals for expanding their capacity to know the present availability of supports and programs ranged from low to high technology solutions. As the suggested technologies advanced along the spectrum, they moved from general awareness of available services at the low tech end, to specific in-the-moment tactical suggestions at the high end. At the low end of the spectrum, they imagined “resource people,” available by phone, to “point me in the right direction at the start of the caregiving journey” (**SR65**). Others imagined this sort of service navigation to extend beyond an initial consultation to offer tailored suggestions along the care journey. As one FC described:

“[I] want to phone someone up and say: “Okay. I’m having issues with this. What do I do?” Then [they would] help you through it. **FG3R8**

At the higher end of the spectrum of technology solutions, FCs imagined advanced search engines, and potentially artificial intelligence, organizing and triaging an overabundance of information available on the Internet. One FC aptly summarized the current state of affairs:

There is too much information and not enough time to make that information utilizable. There are so many websites, apps, checklists etc etc etc (I can't add too many etc.’s). It is like a library with the books in a pile on the floor and no card catalogue to even let me know where to start. **SR50**

The solution to this chaos was, for many FCs, the creation of an online, context-sensitive ‘card catalogue’ or search engine:

You create this library of [caregiving tactics]. You go to your phone and say: “I can’t get my dad in the shower. He’s ready to punch my eyes out. What I tried a month ago ain’t working.” So the library gives you [a tip or trick. Maybe it asks you:] “Did you put on your detective hat today? … Did you ask [yourself] why he’s behaving that way?” **FG6R2**

Another FC emphasized a similar in-the-moment nature of the technology solution she was imagining. Less interested in being challenged to ‘put on her detective hat’ and more interested in immediate tactical support, she noted:

“[It] would be amazing if like the Alzheimer’s Society could program [the app] to give us all [sorts of] different ideas.” So we could say [to our phone]: “Okay. Today Dad doesn’t want to get in the shower. Help! What do I do? I’ve done this. I’ve tried this. I’ve tried this.” Then [the app would] talk to us while our hands are full. **FG6R3**

In these ways both lower tech help phone lines and higher tech search engines and interactive apps were imagined as supports for the broader goal of improving care through greater knowledge of available supports and in-the-moment tactics and solutions to pressing problems.

### Safeguarding the capacity to care

FCs also sought to safeguard their own well-being, and with it, preserve their capacity to care. Their goal here was to improve their resilience and so the sustainability of the care they provided. To become more resilient, FCs sought to gain and hone their own self-care skills that included coping and self-knowledge or self-awareness, as well as opportunities for mentorship, and socialization.

One FC described the search for balance in the following terms:

We focus on the negative all the time. We’re always concentrating on what’s going wrong because we want to fix it. [Focusing on] … the positives sort of evens it up. Even if it’s just for 10 minutes or an hour or something like that, then [I could] say to my friend: “Yeah. I had a bad day, but today is better. Guess what? I actually deflected my mum, and got her to comply.” You’ve got to take your small victories, and I think you have to celebrate them because there’s not a lot. **FG10R3**

This FC describes making herself more resilient by rebalancing the negative and positive aspects of her care work. Finding a way to celebrate the small victories shifted the balance in her focus and thereby avoided immediately anticipating the next negative situation. As resilience flows from focusing on the positive, the care work itself becomes more sustainable. For another FC, finding balance involved finding a way to “handle [the caregiving experience] mentally and emotionally so that I can transform that into effective caregiving for my family and for the one that I’m looking after.” **FG7R2.**

FCs also tended to focus on finding more time and space for themselves as part of their overall sustainability. Specifically, they sought “more down time,” (**SR230**) “more hours in the day to care for myself,” (**SR43**) and the ability to “care for my own health needs while caring for others” (**SR6**). These extra moments “just for me” (**SR60**), “as a person with my own interests and needs” (**SR195**) would be used to “ride my bike,” (**SR136**), “read, relax, have a massage” (**SR141**), “take a vacation with my husband so we can actually spend time together,” (**SR264**) and to “‘vent’ my emotions,” (**SR54**).

One FC described what she saw as a causal relationship between taking time off and becoming more resilient:

If caregivers had time off, their health would hopefully remain good. Currently, burnout and health implications are inevitable. **SR264**

As well as seeking time for themselves “to focus on mindfulness [and] reduce stress and anxiety” **(SR215)**, FCs aspired to also have supportive relationships and resources. This is to say, theirs was not an exclusively inward turn to find peace of mind, but also a reaching out to others to find “mediators/facilitators to help our family members when emotions and accusations run high” **(SR47).** These could be mental health professionals who helped FCs “understand that I don’t need to feel guilty when I put my own needs first occasionally” **(SR64),** or other caregivers and friends who could be mentors and sounding boards **(SR89, SR146)**. One FC described joining:

a café where it’s a drop-in, where [caregivers] come together. It’s more of a party atmosphere in that, you know, they’re not just sitting there crying. They’re enjoying life. **FG1R4**

In these ways FCs seeking to safeguard their capacity to care turned both inwards to reduce stress and harness positive energy, and outwards to engage with peer mentors and form a community with others experiencing similar care journeys.

FCs described using already available technological supports to achieve their goals. One FC described using off-the-shelf technology to support the inward turn required to find serenity, acceptance, and positivity in the face of difficult days:

My [Smart] watch tells me to breathe. It tells me to stand. Honestly. Sometimes it gets me up, and I sit and I take a moment. I breathe. I think that actually works for some people. Maybe [a modification of this could be to offer prompts] like: “Hey - don’t forget about your alone time!” **FG4R2**

As with the majority of the technology suggestions we encountered, FCs envision a modification of existing technology. Rather than simply setting off a reminder to ‘stand and breathe’ that she would have programmed herself, she imagines the smart watch proactively suggesting calming or stress reducing activities. Others suggested the watch could take its cues about when to prompt relaxation and alone-time from monitored personal information like blood pressure or heart rate. An elevated rate would prompt a suggestion to ‘take some me time.’

If these were the preferred technological responses to turning inward for respite and self management, FCs also imagined technology supporting turning outward to others by connecting them to mentors, counsellors, and peer supports. A FC described technology that would make it possible to:

walk around the house with my earbuds in and I’m getting counselling here, I’m getting coached, you know? I would choose [the topics I was hearing about] from a menu or something, like an index. **FG10R3**

Another FC noted that the content of these archived counselling and coaching sessions would likely come from altruistic community members and not commercial technology developers.

I mean, people, they’re not looking to make money. They’re compelled to share because they have a loved one, and they have an empathetic connection to [other FCs]. They want to give back, just like we do, and it’s free. It’s absolutely free. Rather than go to Google and all the stuff that Google tries to put together, this [would be] a library that’s specific to caregiving for a loved one. **FG6R1**

Here we see both content and its indexing imagined as products of the FC community rather commercial activity. This distinction between the moral motivations of content creators was important for FCs as it rendered the content itself more or less credible and trustworthy.

Other FCs sought to combine counselling and mentoring content with in-person rather than virtual relationships. Rather than imagining a library stocked with the expertise and wisdom of their peers, these FCs sought direct access to those peers and the sense of community that such access would bring. Technology, in these scenarios, was not merely a repository, but also a social utility. As one FC described it, he was looking not only for more information, but also for “better networking with other caregivers, sharing our stories to make things easier” **SR128.** Another FC imagined an online portal, that “… would have a list of support groups or stuff that you would go to” **FG4R3.** Yet another FC was more explicit about the primary purpose of the smartphone application she was imagining. She described completing a profile for an app that she hoped would connect her to others and reduce the social isolation frequently experienced by caregivers.

“Wouldn’t it be great if there was an app [where you filled in:]

‘I’m a caregiver;’

‘I have a person who is this age, this gender;’

‘I’m looking for other people to hang out with.’

Like play dates! How can we find these people? Because I’m sure there are people [just] a block away [from me], who are staying home alone all day. Well, let’s get together!” **FG4R2**

## Discussion

Our findings suggest the need for a paradigm shift in which caregiving is reframed away from the current ‘burden of care’ approach, and towards a focus on sustainability and resiliency. This shift is important given the increasing reliance on FCs to deliver health and social care in the community and in response to advocacy organizations’ calls for caregiving work to become more sustainable [62–64].

A capacity-enhancing goal of FCs was to achieve a more integrated and comprehensive knowledge of existing support services. Participants in our research pointed to the fragmentation of support services, and the navigation and access challenges this fragmentation introduced. The challenges here are likely manifold, resulting from some combination of: 1) diversity in FCs’ needs and goals [25, 65]; 2) diversity within the sectors providing support services to FCs [66, 67]; and 3) the complexity and fragmentation of the health and social security ‘system’ more broadly [67–69]. It is well-documented that FCs who have access to social support services cope better with caregiving stress [70]. The evidence further suggests not only that the use of support service early in the caregiving journey helps FCs sustain their care work longer, [71] but that utilization of social support services results in more positive attitudes, better mental health, improved subjective well-being, and better physical health [29, 72]. This is to say, the utilization of social services and supports is strongly correlated with improved resilience and so sustainability. Despite these positive impacts, most FCs do not, or are unable to, access social and community support services. Utilization rates range from 4.8 to 14.0% and are particularly low among those providing care to people with Alzheimer’s and other dementias [73, 74].

In light of these findings and the range of low and high technology suggestions made by FCs in our study, increased policy and production attention to technological and social solutions to the fragmentation of the support services system is imperative [69]. While our participants envisioned technology that had the potential to improve the care FCs provided by giving greater knowledge of available support services and in-the-moment tactics to manage challenging behaviors, the present state of commercial development has focused on addressing specific challenges in caregiving such as emotional support and education [40, 44, 45] as well as specific physical challenges (e.g. mobility aids, monitoring devices) [75, 76]. Future research and development needs to investigate how technology can be harnessed to respond directly to FCs goals, and not the assumptions product designers make about them. Future technology research needs to investigate how fragmented support services might be better integrated to support FCs through the creation of advanced, online context-specific search engines as well as algorithmic learning services such as Huddol or user-directed assistance applications and platforms (e.g. Google Assistant, Amazon Alexa). Our findings also suggest the credibility and trust development challenges that both for-profit, and not-for-profit, peer support platforms may encounter as they seek to build online communities of FCs, and support one-to-one coaching.

Caregiving responsibilities are associated with a variety of stress-related outcomes [77] that include burnout, chronic health problems, deteriorating self concept, and decline in emotional well-being [78–80]. A recent review shows caregiving for a person with dementia is significantly associated with psychological stress and physical ill-health [29] and that specific features of Alzheimer’s disease such as aggression, sleep disturbance, and depression negatively impact the physical and mental well-being of FCs [81]. The degree to which FCs can successfully adapt to stressful conditions is arguably a function of interactions among a number of factors including caregiving overload, loss of self amidst care responsibilities, lack of social support, and inadequate coping strategies (e.g. problem-solving skills) [70]. Improving FCs’ resilience by mitigating these factors, was central to achieving FCs’ goals of enhancing their caring capacity. Their goal of achieving foreknowledge was at once focused on delivering better care and gaining peace of mind that would make caregiving sustainable. Similarly, they imagined different strategies for adapting to stressful conditions including seeking time for themselves (inward turn to find peace) and developing better coping skills, as well as seeking opportunities for mentorship and socialization. For these goals, and the technology options associated with achieving them, FCs’ sought to enhance their resilience through a dynamic process of positive adaptation [82, 83]. Self-development skills such as coping strategies focused on self-compassion (i.e. balanced awareness of thoughts and feelings or being kind to oneself when things go wrong) and mindfulness (i.e. ability to pay attention to any experience with equanimity) are associated with higher resilience as well as positive outcomes such as life satisfaction, hope, quality of life, and self-esteem for FCs [29, 84, 85]. As our data show, FCs’ motivations in seeking to safeguard their ability to care underscored a desire to explore meanings and values, and to live a meaningful, purposeful, and full life within their existing care responsibilities [86]. One school of thought suggests this sort of growth is, like sustainability, an outcome and consequence of resilience in which FCs gain a greater understanding of their capacities and learn from stressful experiences and active efforts to cope with them [87, 88].

Building on the discussion so far and drawing on other caregiving studies and in-depth findings from our case study, we suggest that a paradigm shift is needed to reframe caregiving from a current deficit frame (burden of care) towards a more empowering frame (resilience that leads to growth and sustainability). The fact that FCs are seeking mechanisms to expand and safeguard their caregiving capacities reflects this shift from a traditional focus on the ‘burden of care’ towards a more resilient model of caregiving. This shifts caregiving as an experience centred on failures and limitations to one focused on personal growth, and balance of positive and negative aspects that flow from being resilient towards more sustainable care.

### Study limitations

There are a range of limitations associated with focus groups. These include: 1) the potential domination of discussions by a few participants [89, 90], 2) social desirability bias, and 3) acquiescence bias. We adopted proactive strategies to mitigate these limitations. To minimize the potential that a few voices would dominate and others would be underrepresented, the lead author moderated all focus groups, solicited a range of views and experiences, guided the discussion, and ensured all participants had an equal opportunity to contribute [48]. To mitigate social desirability bias [91], we built a ‘safe space’ by assuring participants that their responses would remain anonymous and would be held in confidence [91]. To minimize acquiescence bias [92] we avoided yes/no questions and assured participants at the beginning of each focus group that there were no correct answers to the questions we were raising, and their views and experiences were of great importance. Moreover, the two researchers participating in the focus groups managed their questions and comments to ensure their respective positions remained neutral. Prior to convening the focus groups we engaged with FCs through a number of email exchanges to build a shared understanding of the research project and to develop trusting relationships. These email exchanges encouraged relaxed interactions and helped participants to see the research team as familiar and trusted facilitators rather than authorities demanding deference.

## Conclusions

Our research investigating FC’s goals and the role of technology in addressing those goals revealed important insights with implications for policy and practice. The fact that FCs are seeking strategies to enhance and safeguard their care capacities means they are approaching their unpaid work from the perspective of sustainability. As with other evidence in the literature, our study clearly indicates not just a desire among FCs to continue in their caring role, but a belief that supports will help them do just that. FCs’ goals and technology-driven suggestions imply a shift from the current framing of caregiving as a burden towards a frame of growth, resilience and sustainability. Our case study findings show that technology can assist in fostering this resiliency, but that it may well be limited to the role of an intermediary that connects FCs to information and others. As ours is one of a limited number of studies in the field, its findings may not transfer outside of Canada. With this in mind, we call for further research in different institutional and cultural settings. With increasing emphasis given to resiliency and sustainability in caregiving in national and international policy discourses, it is important that comparative knowledge be developed.

## Data Availability

In accordance with our approved research ethics protocol, the focus group transcripts, survey responses, and data analyses generated during the current study will not be publicly available in order to preserve participants’ anonymity.

## Abbreviations

CARP: Canadian Association of Retired Persons
FC: Family carer
FG: Focus group
SR: Survey

## Footnotes

1 The abbreviations SR and FG stand for Survey and Focus Group respectively; these codes are used to protect the anonymity of our research participants.
